# Centipede assemblages along an urbanization gradient in the city of Heraklion, Crete (Greece)

**DOI:** 10.3897/zookeys.510.8414

**Published:** 2015-06-30

**Authors:** Gabriella Papastefanou, Eleni Panayiotou, Moisis Mylonas, Stylianos Michail Simaiakis

**Affiliations:** 1 Department of Biology, University of Crete, GR-71100 Heraklion, Crete, Greece; 2 Natural History Museum of Crete, University of Crete, Knossos Av., PO Box 2208, GR-71409 Heraklion, Crete, Greece

**Keywords:** Abundance, activity density, *Eupolybothrus litoralis*, *Lithobius nigripalpis*, pitfall traps, *Scolopendra cretica*, *Scutigera coleoptrata*, spatial distribution, species diversity, temporal distribution

## Abstract

Global urbanization is a major force that causes alteration and loss of natural habitats. Urban ecosystems are strongly affected by humans and there is a gradient of decreasing human influence from city centers to natural habitats. To study ecological changes along this continuum, researchers introduced the urban-rural gradient approach. The responses of centipedes to an urbanization gradient (urban-suburban-rural areas) were studied using pitfall traps in and near the city of Heraklion, in the island of Crete, Greece, from November 2010 to November 2011. Our results do not support the intermediate disturbance hypothesis, in which suburban areas located in the transitional zone between urban and rural habitats failed to indicate significant increase in terms of species richness and diversity.

## Introduction

With respect to global threats, some scientists believe that our planet is facing a new biodiversity crisis, frequently called as the sixth mass extinction, which is a human-caused phenomenon (Eldredge 1998). Regarding biodiversity, over the past few decades there has been a growing interest to explore biological components of cities in order to understand their ecology at different geographical scales (McDonnell et al. 2009). At the same time, urban areas are increasing worldwide (Sorace 2001), while the rapid and worldwide urbanization of human population (Niemelä 1999) raises concerns about the sustainability of cities (Andersson 2006).

A city represents an ideal terrestrial ecosystem to investigate fauna composition. It retains specific microclimatic and hydrological parameters and is sensitive to human activities and climate change (Ricklefs 1990). In addition, many invertebrate species form a biodiversity component critical for the persistence of the ecosystem (Oliver and Beattie 1996), and are well-adapted to live in this environment (Wallwork 1976). However, soil invertebrates are marginalized from conservation works, mainly because of lacking data (Ward and Lariviere 2004). Consequently, without information on species distribution, community ecology and on the evolutionary processes that influence them, terrestrial invertebrates may remain threatened, leading to loss of species. In fact, habitat fragmentation, habitat loss, accumulation of pollutants to soil, water and atmosphere affect negatively native biodiversity (Clark et al. 2007) and are primary causes of species extinctions (e.g., Wilcove et al. 1998, McKinney 2002).

Nowadays thousands of species are characterized as nationally extinct, threatened, or near threatened in broad habitats, particularly in urban areas, as a result of the significant declining areas of natural patches. These habitats represent an important patchily distributed environment for thousands of species (Hanski 2004). For example, it is well known that in highly fragmented landscapes (e.g., city), one decaying tree trunk or a flower-bed may support local soil populations for many generations. Soil arthropods such as ground beetles, centipedes, millipedes, spiders, and scorpions can be readily surveyed. Regarding urban areas, species richness is often determined by climatic changes, solar radiation, and the availability of host plants. Thus, soil arthropods are potentially useful ecological indicators of urbanization (Clark et al. 2007).

The effect of urbanization on biodiversity has focused primarily on vertebrates (e.g., Germaine et al. 2001). Several works have also investigated terrestrial invertebrates (e.g., ground beetles) along a gradient from a highly disturbed urban environment to a less disturbed rural environment (e.g., Alaruikka et al. 2002, Niemelä et al. 2002, Ishitani et al. 2003, Ulrich et al. 2008). In general, in northwestern parts of Europe such focused studies are numerous, but in southeastern Mediterranean region the knowledge is scanty.

Because of the relatively high diversity and the quite high availability of species occurrence records, centipedes provide a suitable taxonomic group for studying ecological aspects. However, only few studies have focused on the impact of urbanization on centipede species assemblages. In this study we performed several comprehensive analyses to investigate the responses of centipede species in the city of Heraklion, on Crete. In particular:

- we quantify species richness, abundance and diversity along an urban-rural gradient,
- we study centipede species structure emphasizing on the spatial and temporal distribution along the three urbanization zones, and,
- we explore patterns of distribution of the identified generalist species.

## Methods

### Study area

The city of Heraklion (35°20'0"N; 25°8'0"E) is the largest city of Crete and the fourth largest in Greece located in the centre of the northern coast of the island. It covers an area of approximately 24 km^2^ with an estimated population of 173,450 (according to the General Population Census 2011) at a density of 7,227 residents per square kilometer. Heraklion is mainly flat with several prominent hills, characterized by numerous floral species, mainly introduced, such as *Hirschefeldia incana* and *Conyza albida* (common species along the roadsides), *Petromarula pinnata* (endemic plant of Crete), *Ailanthus altissima* (dominant plant at the archaeological site of Knossos), *Hyoscyamus aureus* (common in the Venetian city walls) (for further habitat description see also Vogiatzakis and Rackham 2008 and Kaltsas et al. 2014).

**Figure 1.**
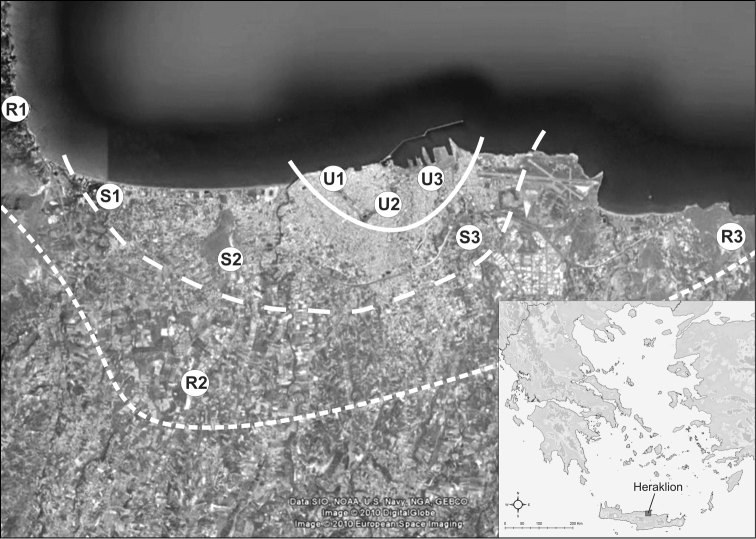
Map of the study area and sampling sites in and near Heraklion city. **U** urban sites (**U1** athletic centre, roads, parking area, buildings and gardening activities **U2** roads, parking area, dense buildings and gardening activities **U3** Heraklion port, roads, numerous buildings and gardening activities) **S** suburban sites (**S1** hotels, roads, sandy substrate **S2** hotels, electricity power factory, roads **S3** industrial area, roads, numerous buildings) **R** rural sites (**R1–R3** roads and little grazing).

We selected three sampling areas along an urbanization gradient, as proposed by the Globenet protocol (Niemelä et al. 2000), from north to south and from west to east of Heraklion: a) a highly disturbed habitat – urban, b) a moderate disturbed habitat – suburban, and, c) a less disturbed habitat – rural. Within each disturbance area we settled three replicate sites covering the west (1), the south (2) and the east (3) side of the city (Fig. 1). In particular, nine sampling sites were selected: i) three urban sites within the city of Heraklion, ii) three suburban sites on the boarders of the city, and iii) three rural sites in natural environment. The selection of the sampling sites was made on the basis of the similarity of vegetation and the percentage of built-up area (Ishitani et al. 2003, Kaltsas et al. 2014). Further details on the environment of each site can be found in Fig. 1. The urban habitats, at the western part of the Venetian city walls (U1), at the southern side of the Venetian city walls (U2), and at the eastern part of the city, not far from the main port (U3), were characterized by the dominance of nonnative herbaceous vegetation. The suburban habitats were located at the western end of Ammoudara district (S1), at the southern part of the city near the road to Moires (S2), and at the eastern side of the city close to the industrial area (S3), and were dominated by indigenous herbaceous vegetation and *Nerium oleander*. Rural habitats were located in the west, at the Palaiokastro bridge next to the national road (R1), in the south district of Heraklion (R2), and in the east near Kokkini Chani close to the national road (R3). The Euclidian distances between the sampling sites were: U1-U2 = 1.22 km, U2-U3 = 1.2 km, S1-S2 = 4.0 km, S2-S3 = 6.4 km, R1-R2 = 6.99 km, R2-R3= 13.21 km. All rural habitats were dominated by typical phrygana, such as *Sarcopoterium spinosum*, *Thymbra capitata* and *Genista acanthoclada* (see also Kaltsas et al. 2014). We could not settle any sampling site in the north side of Heraklion because of its proximity to the Aegean Sea.

### Sampling design

Centipedes were collected with pitfall traps along the aforementioned urban–rural gradient in accordance to the GLOBENET program protocol for capturing soil arthropods (Niemelä et al. 2000, 2002). At each site we placed 10 traps along a transect line positioned at a distance of 10 m from one another and specimens were collected monthly. Each trap was a plastic container with a diameter of about 10 cm placed into the ground in a depth of almost 12 cm. Overall we placed 90 traps across the urban – rural gradient. Pitfall traps contained ethylene glycol as a preservative liquid. The collection of material took place over a year, from November 2010 to November 2011. We determined geographic coordinates for each sampling site using a GPS apparatus. All captured centipedes are preserved in 95% alcohol and are deposited in the Myriapod collection of the Natural History Museum of Crete (hereafter NHMC).

### Data analyses

Centipede species richness for each site was estimated using two nonparametric richness estimators, in particular Chao1 and Bootstrap (see Colwell et al. 2012 for review), based on species-by-sample data. We calculated survey completeness for each sampling site as the observed number of captured species divided by the average estimated number of species. In agreement with Meijer et al. (2011), a value of sampling completeness above 0.75 is generally accepted. In our case, none of the nine mentioned sites were excluded from analyses. We also prepared species accumulation curves, as a function of sampling completeness, of the rate at which new species were found at each site along the urbanization gradient.

In terms of richness the number of species and genera was counted at each site. We also calculated the diversity of centipedes using: (i) the Shannon diversity index *H*’ (for further details see Magurran 1988), and (ii) the evenness diversity index *J*’ (for further details see also Magurran 1988).

We calculated activity density in terms of number of individuals per 100 trap-days at each habitat along the gradient. The temporal distribution of centipede assemblages was analysed in terms of the average number of species per sampling period, i.e., one month, called ᾱ diversity, and the proportion of cumulative *α* diversity, known as a measure of temporal turnover (Romanuk and Kolasa 2001):


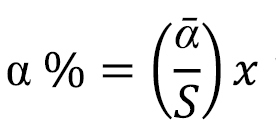


where *S* is the total number of species captured in a site (Zamora et al. 2007). We also measured the temporal beta diversity (*β*t), known as the temporal change of species structure at each site. To find temporal beta diversity, we first calculated the complementarity index for each sampling period, i.e., one month (see Colwell and Coddington (1994):


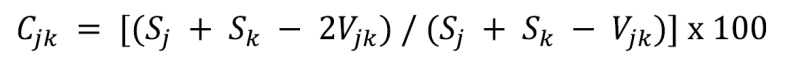


where *S_j_* is the number of species captured in sampling period *j*, *S_k_* the number of species captured in consecutive sampling period *k* (k=j+1) and *V_jk_* the common species captured in periods *j* and *k*. After these calculations, temporal beta diversity (*β*t) was measured as the average for each site assemblage.

Among others, we performed one-way ANOVA tests for differences in richness, abundance, activity density, and diversity (Shannon *H*’, temporal diversity, *β*t and proportion of cumulative *α* represented by average *α*, α %) along the urbanization gradient.

We also performed a non-metric multi-dimensional scaling (NMDS) ordination plot based on Bray-Curtis dissimilarities of square-root transformed centipede abundance data, to find out whether there are structural differences in centipede assemblages along the urban–rural gradient. Structural differences may concern species composition and species activity density. NMDS analysis was performed in PAST 2.16 (Hammer et al. 2001). Furthermore, we tested if temporal beta diversity (*β*t) increases as α % decreases using a simple linear regression.

## Results

Overall, 993 individuals (36.3 individuals per site / 100 trap-days) were collected and identified, belonging to 18 centipede species (8.0 ± 1.7 per site) and 11 genera (5.9 ± 0.9 per site) (Table S1 in Suppl. material 1). Seven species were collected at all three urbanization levels (*Clinopodes flavidus*, *Eupolybothrus litoralis*, *Lithobius creticus*, *Lithobius erythrocephalus*, *Lithobius nigripalpis*, *Scolopendra cretica*, *Scutigera coleoptrata*). Among these, *Lithobius creticus* and *Scolopendra cretica* are endemic in Crete. Six species were characterized as single habitat species (e.g., *Cryptops trisulcatus*, *Lithobius aeruginosus*, *Lithobius lapidicola*, *Lithobius pamukkalensis*, *Pachymerium ferrugineum*, *Schendyla nemorensis*), out of which four, namely *Cryptops trisulcatus*, *Lithobius aeruginosus*, *Lithobius lapidicola*, and *Schendyla nemorensis*, were represented by single individuals (singletons) (see Table S1 in Suppl. material 1). The most abundant species were *Scolopendra cretica*, *Lithobius nigripalpis*, *Scutigera coleoptrata*, and *Eupolybothrus litoralis*, with approximately 37.7%, 21.0%, 17.9%, and 17.3% of the total centipede catch, respectively.

**Figure 2.**
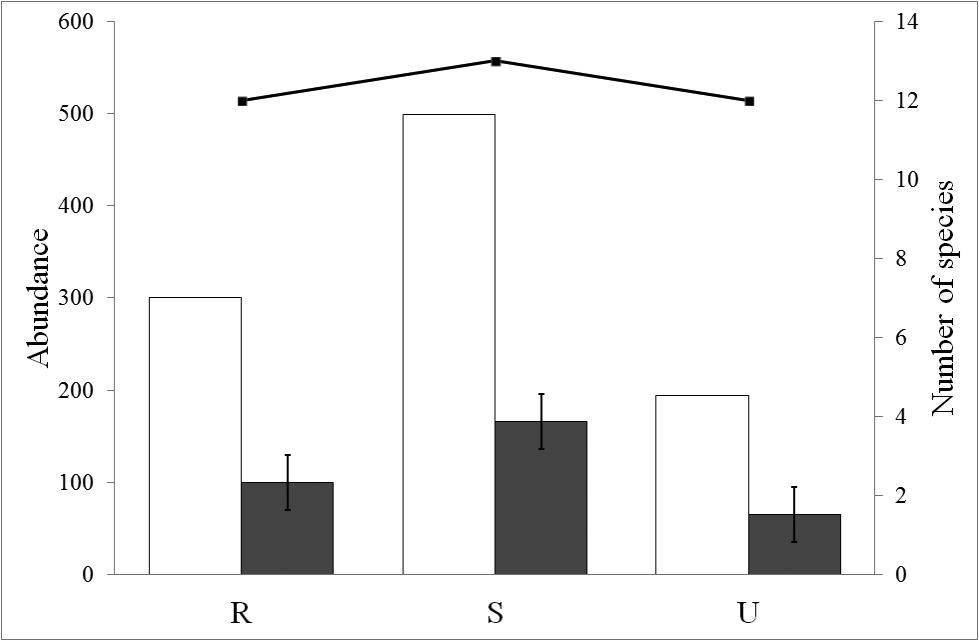
Total and average abundance of centipedes as well as total species richness along the urban-rural gradient. White columns show total abundance, grey columns show average abundance with bars with standard deviation, dark line shows species richness.

**Figure 3.**
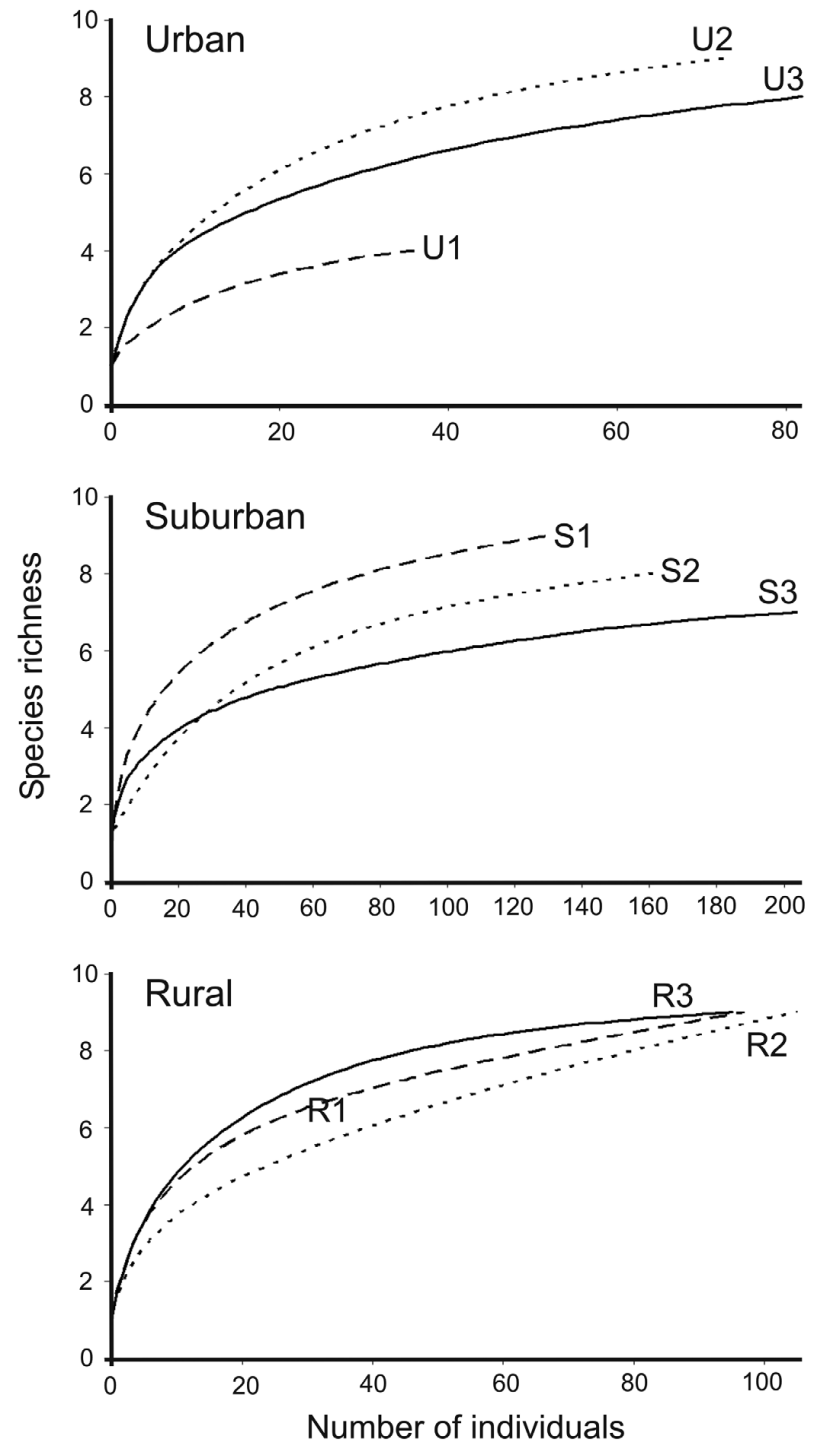
Species accumulation curves (recorded as a function of sampling effort) of the rate at which new species are found within each site along the urbanization gradient.

The total and average abundance were maximal in suburban sites, minimal in urban sites, and intermediate in the rural sites (Fig. 2). As for the species richness, 12 species were identified in both rural and urban habitats, while 13 species were collected from the suburban sites (Fig. 2). In detail, most of the individuals were captured in suburban (499 individuals, 50.3% of the total) and rural habitats (300 individuals, 30.2% of the total), while in the city centre, only 194 centipede individuals were collected (19.5% of the total) (Table 1). Shannon and evenness diversity indices were slightly different along the urbanization gradient (Table 1). The species accumulation curves of the rate at which new species are found within each site along the urbanization gradient are presented in Fig. 3. Accumulation curve data showed that completeness of samplings was relatively high for all nine sites, ranging between 0.79 and 1.00, with an average value of 0.91 ± 0.06 per site (Table 1).

**Table 1.** Summary results of centipede species diversity for each site (R: rural, S: Suburban, U: urban). Richness is shown in terms of captured genera (G), species (S), total number of individuals in samples (Sa). Species diversity is estimated based on Shannon-Wiener (*H*’) and Evenness (*J*’) diversity indices. Sampling effort is shown in terms of expected number of species (Se) based on Chao 1 and Bootstrap estimators (mean ± SD) and completeness of samplings (Sc). Temporal diversity is shown in terms of activity density (A), average species richness (ᾱ), proportion of cumulative *α* represented by average *α* diversity (α%), and temporal beta diversity (*β*t).

Site	G	S	Sa	H’	*J*’	Se Chao 1 (mean ± SD)	Se Bootstrap (mean ± SD)	Sc	A	ᾱ	α%	βt
U1	4	4	37	0.72	0.51	4.12 ± 0.72	4.48 ± 0.29	0.93	12.14	1.17	29.17	62.96
U2	6	9	74	1.61	0.56	9.77 ± 2.16	9.98 ± 0.05	0.91	25.68	2.25	25.00	60.15
U3	7	8	83	1.55	0.59	8.54 ± 1.38	8.85 ± 0.28	0.92	25.66	2.67	33.33	57.27
S1	6	9	132	1.51	0.50	10.00 ± 2.26	9.85 ± 0.02	0.91	43.93	3.25	36.11	49.85
S2	5	8	162	0.75	0.27	9.00 ± 2.25	9.05 ± 0.03	1.00	53.30	2.33	29.17	60.30
S3	6	7	205	1.17	0.46	7.35 ± 1.22	7.68 ± 0.09	0.93	66.35	3.00	42.86	45.61
R1	6	9	98	1.62	0.56	12.00 ± 4.48	10.07 ± 0.33	0.82	31.58	3.00	33.33	71.04
R2	7	9	106	1.31	0.41	12.51 ± 4.74	10.56 ± 0.27	0.79	33.95	2.42	26.85	74.39
R3	6	9	96	1.68	0.60	9.37 ± 2.09	9.51 ± 0.27	0.95	33.69	3.17	35.18	42.88

The activity density (individuals/100 trap-days) of centipede assemblages ranged from ca 12.1 to 25.7 in urban habitats (mean value of 21.2 ± 7.8), from ca 43.9 to 66.3 in suburban habitats (mean value of 54.5 ± 11.3), and from ca 31.6 to 34.0 in rural sites (mean value of 33.1 ± 1.3) (see Table 1 and Table S2 in Suppl. material 1). The average species richness (ᾱ) varied from ca 1.2 to 3.3 among the sampling habitats (Table 1). Moreover, the proportion of cumulative *α* represented by average *α* diversity (α %) varied from 25% to about 43% and the temporal beta diversity (*β*t) varied from about 42.9 to 74.4 among the sampling sites (Table 1).

The difference in species richness along the urbanization gradient was not statistically significant (Table 2). Likewise, although the average abundance of centipedes in suburban sites was about 1.7 and 2.6 times higher than in rural and urban sites respectively, we did not find any statistical significance (Table 2). Though the mean value of Shannon diversity index (*H*’) was higher in the rural sites (1.53 ± 0.2) than in the urban (1.29 ± 0.5) and suburban habitats (1.15 ± 0.4), it did not differ significantly among the three areas (*F* = 0.27, *p* = 0.77). In contrast, activity density differed significantly along the gradient (*F* = 4.47, *p* = 0.02), as a result of the significantly lower values of A density in urban sites (21.2 in average) compared to suburban (54.5 in average) and rural sites (33.1 in average). Moreover, as shown in Table 2, the proportion of cumulative *α* represented by average *α* diversity (*α* %) is significantly smaller in urban sites compared to rural and suburban areas (*F* = 3.09, *p* = 0.03). However, the difference in temporal beta diversity (*β*t) along the gradient level was statistically insignificant (Table 2).

**Table 2.** One-way ANOVA results showing statistical differences of species richness, abundance, activity density (A density), and diversity (Shannon *H*’, temporal diversity, *β*t and proportion of cumulative *α* represented by average *α*, α%), along the urbanization gradient in Heraklion. Degrees of freedom (DF), sum of squares (SS), mean square (MS), F values (*F*). The last column shows the significant differences between the gradient levels (p < 0.05) based on the Tukey test. R: rural, S: suburban, U: urban.

	DF	SS	MS	F	p	Tukey test
Species richness	2	12.39	6.19	2.39	0.11	
Abundance	2	2664.11	1332.06	0.91	0.41	
Shannon *H*’	2	2.70	1.35	0.27	0.77	
A density	2	47.65	23.82	4.47	0.02	U < R, S
*β*t	2	653.45	326.72	0.96	0.39	
*α*%	2	5.56	2.78	3.09	0.03	U < R, S

**Figure 4.**
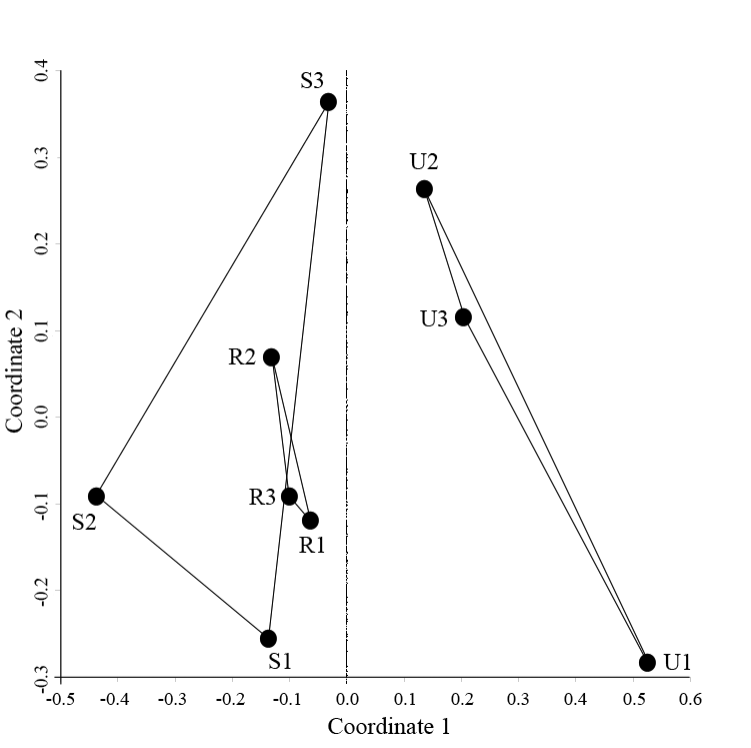
NMDS two-dimensional ordination plot based on the Bray-Curtis dissimilarity matrix of the nine sites along the urbanization gradient.

**Figure 5.**
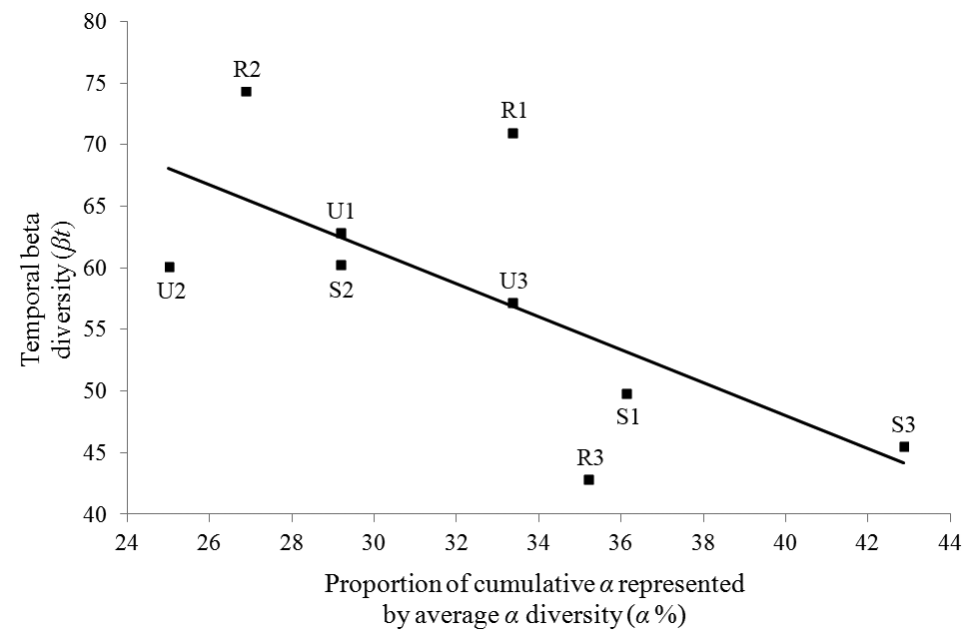
Simple linear regression between temporal beta diversity (*β*t) and the proportion of cumulative *α* represented by average *α* diversity (*α*%). Model: (*β*t) = 101.65 – 1.34(*α*%), *r*² = 0.47, *p* = 0.04. U: urban sites, S: suburban sites, R: rural sites.

The structural difference of urban centipede assemblages compared to the other two habitat zones was apparent when we performed NMDS. The two-dimensional (2D) ordination plot explained 90% of the variance in the distance matrix (Axis 1: 72 %, Axis 2: 18 %, stress = 0.12; see Fig. 4). As expected, temporal beta diversity (*β*t) was independent of species richness (Spearman’s *r_s_* = 0.05, *p* = 0.89). Furthermore, *β*t decreases significantly (*r*^2^ = 0.47, *p* = 0.04) as α% increases (that is, the difference between cumulative and average α decreases) (Fig. 5).

**Figure 6.**
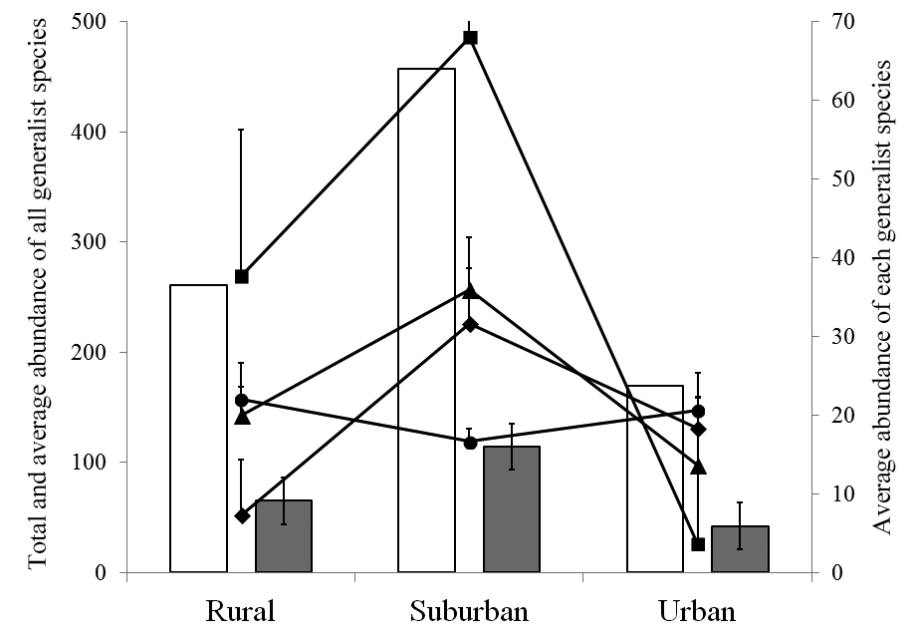
Total and average abundance of all four generalist species as well as average abundance of each generalist species along the urban-rural gradient, *Eupolybothrus litoralis* (♦), *Lithobius nigripalpis* (▲), *Scolopendra cretica* (■), *Scutigera coleoptrata* (●). White columns show total abundance, grey columns show average abundance with bars with standard deviation.

*Scolopendra cretica* was the dominant species in rural and suburban sites covering 50.6% and 86.9% of the total captures in these sites respectively. However, in urban sites its capturing coverage lowered to 2.1%, while the dominant species in urban sites was *Scutigera coleoptrata* (72.7%). *Scutigera coleoptrata* also covered a large portion of captures in rural areas (21%). Two more species could be considered dominant, namely *Lithobius nigripalpis* that covers 14.1% and 18.5% of the total captures in urban and suburban sites respectively, and, *Eupolybothrus litoralis* that covers 25.5% and 19.8% of the total captures in urban and suburban sites respectively. *Lithobius nigripalpis* was also dominant in rural areas covering 12.8% of the total captures. The percentage of individuals of opportunistic centipede species to the total individuals proved to differ significantly along the urbanization gradient. The abundance of opportunistic species in the suburban sites was 1.75 and 2.7 times higher than their abundance in rural and urban sites respectively (Fig. 6). Generalist species covered 91.6% of the total captures in suburban areas, whereas the respective percentages in rural and urban sites were 87% and 55%. Specifically, all generalist species were mostly abundant in suburban sites, except for *Scutigera coleoptrata* which in this area appeared to be less abundant.

## Discussion

According to several studies, urbanization reduces species richness in many animal groups owing to the impoverished flora, in terms of habitat loss (McKinney 2002) and habitat fragmentation leading to isolated populations (Collins et al. 2000). In addition, studies have shown that the urban soil content in nitrogen and carbon is profoundly lower compared to the rural soil either owing to their cycle alteration (Lorenz and Lal 2009) or as a result of the impenetrable surfaces in urban areas (Raciti et al. 2012).

Unlike the aforementioned examples, our results failed to indicate negative urbanization effect on centipede species richness and diversity. Alaruikka et al. (2002) studied carabid and spider species in Finland only to reach the same conclusion on the abundance and species richness, suggesting that, in particular, spiders might be more sensitive to small-scale habitat changes rather than to large-scale changes. Likewise, urbanization has not reduced overall centipede species richness and diversity in the city of Poznaǹ (Leśniewska et al. 2008), nor has reduced the ground beetles species richness in several cases where data from Globenet Project were used (Magura et al. 2010b). Even though abundance in our results, differed along the urbanization gradient with the highest abundance found in suburban areas, followed by the second higher abundance in rural areas, the difference was not confirmed statistically.

Additionally, in terms of species richness and diversity, our results are not statistically significant to support the suburban peak (McKinney 2002). Similar results were also reported about isopods (Hornung et al. 2007) and ground-dwelling spiders (Horvath et al. 2012). In general, suburban environments are considered as transitional zones between natural and urban habitats and show characteristically high environmental heterogeneity, since diverse habitats occur together alongside one another (McKinney 2002). This pattern is well-documented in numerous urban-to-rural gradient studies that examine changes in diversity on plants (Kowarik 1995), butterflies (Blair 1999, Konvicka and Kadlec 2011), mammals, birds, lizards, bumblebees ants (McKinney 2002 and references therein) and carabid beetles (Tothmeresz et al. 2011). On the other hand, Ishitani et al. (2003) found that species richness in carabid beetles increases from urban to rural environments in Japan, similar to the case of Magura et al. (2010a) who studied ground-dwelling spiders along an urban-rural forest gradient in Hungary.

As for temporal beta diversity (*β*t), it showed no difference among the three zones indicating no variance in species richness between sampling periods. The high (*β*t) values found, in most cases above 50, are attributed to nomadic assemblages or degraded habitats, under fast environmental alterations or intense perturbations (Romanuk and Kolasa 2001). In addition, temporal turnover of species assemblages barely changed along the urbanization gradient in the city of Heraklion. However, our results have validated α% as a quantitative metric of temporal turnover as it was inversely proportional to *β*t. These results are in accordance with Moreno and Halffter (2001). With α% values relatively low (see Table 1) and statistically higher in rural and suburban areas (see Table 2), we assume that temporal turnover is greater in urban areas. Moreover, statistically significant lower activity density in the suburban and rural sites compared to the urban sites shows both higher centipede activity and possibly higher population density.

The structure of centipede assemblages differed substantially along the urban-rural gradient. We also observed great similarity in centipede diversity between rural and suburban sites. The distribution from rural to suburban areas is not impossible since in suburban sites human constructions retain green areas as centipede habitats. Within the city of Heraklion though, species composition is significantly different from both rural and suburban due to great habitat loss and fragmentation. The four generalist species (*Eupolybothrus litoralis*, *Lithobius nigripalpis*, *Scolopendra cretica*, *Scutigera coleoptrata*) were found in great abundance in all zones. However, highest centipede abundance was found in suburban areas, followed by the rural areas and fewer individuals were caught in the centre of the city, showing that mild human pressure can promote the abundance of these species. Three species, namely *Lithobius aeruginosus*, *Lithobius lapidicola*, and *Schendyla nemorensis* were found exclusively in the city centre, suggesting that human activities such as gardening and landscaping introduce new species in cities through transferred soil. On the other hand, *Cryptops trisulcatus* and *Pachymerium ferrugineum* occurred only in suburban sites indicating specific habitat preferences under large stones and sand soil substrate, respectively. Although *Scolopendra cretica* was the dominant species in rural and suburban habitats, its capturing coverage was extremely low in urban environments showing low tolerance to the intense human activity. In contrast, *Scutigera coleoptrata* with a capturing coverage of about 73% showed a highly opportunistic character in the city habitats. Finally, only one representative of the species *Lithobius pamukkalensis* was collected in rural sites, which is so far the most western distribution of this species in Crete.

## Conclusion

Different studies consistently highlight alternative animal responses to urbanization. Even same animal groups differed in their reaction to the increasing human activity in different cities. Our study failed to indicate negative urbanization effect on centipede species richness and diversity in the city of Heraklion. It is noteworthy that even though there is a trend of increasing abundance towards the suburban habitats, the difference was not confirmed statistically. Furthermore, our results are not consistent with those that have supported the intermediate disturbance hypothesis. This means that the suburban environment of Heraklion may not be considered as transitional zone between natural and urban habitats.

## Supplementary materials

Supplementary material 1 Abundance and activity density of centipede species per sampling site. Data type: occurrence Explanation note: List of species and individuals captured at each sampling site. This dataset is made available under the Open Database License (http://opendatacommons.org/licenses/odbl/1.0/). The Open Database License (ODbL) is a license agreement intended to allow users to freely share, modify, and use this Dataset while maintaining this same freedom for others, provided that the original source and author(s) are credited.Gabriella Papastefanou, Eleni Panayiotou, Moisis Mylonas, Stylianos Michail Simaiakis
