# Supplementary material for: Centipede assemblages along an urbanization gradient in the city of Heraklion, Crete (Greece)
Source: Zookeys. 2015 Jun 30;(510):163–79. doi: 10.3897/zookeys.510.8414 (PMC4523771; doi:10.3897/zookeys.510.8414)
Supplement: Supplementary material 1 — Abundance and activity density of centipede species per sampling site. [file zookeys-510-163-s001.docx]

Table S1. List of species and individuals captured at each sampling site (R: rural, S: Suburban, U: urban). Species with asterisk are Cretan endemic. Grey cells indicate singletons (single site species).

| Species | R1 | R2 | R3 | S1 | S2 | S3 | U1 | U2 | U3 |
| --- | --- | --- | --- | --- | --- | --- | --- | --- | --- |
| *Bothriogaster signata* (Kessler, 1874) | 0 | 1 | 0 | 0 | 0 | 0 | 0 | 0 | 2 |
| *Clinopodes flavidus* Koch, 1847 | 0 | 2 | 1 | 0 | 0 | 2 | 2 | 6 | 4 |
| *Cryptops hortensis* (Donovan, 1810) | 1 | 0 | 0 | 0 | 0 | 1 | 0 | 0 | 0 |
| *Cryptops trisulcatus* Brolemann, 1902 | 0 | 0 | 0 | 0 | 1 | 0 | 0 | 0 | 0 |
| *Dignathodon microcephalus* (Lucas, 1846) | 1 | 1 | 5 | 0 | 4 | 0 | 0 | 0 | 0 |
| *Eupolybothrus litoralis* (L. Koch, 1867) | 3 | 14 | 5 | 1 | 3 | 91 | 0 | 30 | 25 |
| *Henia bicarinata* (Meinert, 1870) | 0 | 0 | 0 | 4 | 0 | 0 | 0 | 1 | 0 |
| *Lithobius aeruginosus* L. Koch, 1862 | 0 | 0 | 0 | 0 | 0 | 0 | 1 | 0 | 0 |
| *Lithobius creticus* Dobroruka, 1977 ^*^ | 7 | 1 | 5 | 3 | 3 | 0 | 0 | 3 | 1 |
| *Lithobius erythrocephalus* C.L. Koch, 1847 | 7 | 0 | 2 | 3 | 6 | 2 | 0 | 3 | 0 |
| *Lithobius lapidicola* Meinert, 1872 | 0 | 0 | 0 | 0 | 0 | 0 | 0 | 1 | 0 |
| *Lithobius lucifugus* L. Koch, 1862 | 1 | 1 | 0 | 1 | 1 | 0 | 0 | 0 | 0 |
| *Lithobius nigripalpis* L. Koch, 1867 | 26 | 21 | 13 | 13 | 10 | 85 | 0 | 22 | 19 |
| *Lithobius pamukkalensis* Matic, 1980 | 0 | 0 | 3 | 0 | 0 | 0 | 0 | 0 | 0 |
| *Pachymerium ferrugineum* (Koch, 1835) | 0 | 0 | 0 | 11 | 0 | 0 | 0 | 0 | 0 |
| *Schendyla nemorensis* (C.L. Koch, 1837) | 0 | 0 | 0 | 0 | 0 | 0 | 0 | 0 | 1 |
| *Scolopendra cretica* Lucas, 1853 ^*^ | 14 | 60 | 39 | 56 | 134 | 14 | 5 | 2 | 4 |
| *Scutigera coleoptrata* (Linnaeus, 1758) | 38 | 5 | 23 | 40 | 0 | 10 | 29 | 6 | 27 |
| Total | 98 | 106 | 96 | 132 | 162 | 205 | 37 | 74 | 83 |

Table S2. Activity density of centipede species captured at each sampling site (R: rural, S: Suburban, U: urban).

| Species | R1 | R2 | R3 | S1 | S2 | S3 | U1 | U2 | U3 |
| --- | --- | --- | --- | --- | --- | --- | --- | --- | --- |
| *Bothriogaster signata* |  | 0.35 |  |  |  |  |  |  | 0.65 |
| *Clinopodes flavidus* |  | 0.68 | 0.37 |  |  | 0.66 | 0.62 | 2.43 | 1.20 |
| *Cryptops hortensis* | 0.36 |  |  |  |  | 0.36 |  |  |  |
| *Cryptops trisulcatus* |  |  |  |  | 0.42 |  |  |  |  |
| *Dignathodon microcephalus* | 0.29 | 0.28 | 2.13 |  | 1.35 |  |  |  |  |
| *Eupolybothrus litoralis* | 1.28 | 4.62 | 1.71 | 0.31 | 1.00 | 28.55 |  | 9.93 | 8.35 |
| *Henia bicarinata* |  |  |  | 1.32 |  |  |  | 0.37 |  |
| *Lithobius aeruginosus* |  |  |  |  |  |  | 0.31 |  |  |
| *Lithobius creticus* | 2.12 | 0.33 | 1.54 | 1.88 | 1.63 |  |  | 0.94 | 0.33 |
| *Lithobius erythrocephalus* | 2.15 |  | 0.67 | 0.96 | 1.88 | 0.69 |  | 0.94 |  |
| *Lithobius lapidicola* |  |  |  |  |  |  |  | 0.31 |  |
| *Lithobius lucifugus* | 0.35 | 0.31 |  | 0.31 | 0.31 |  |  |  |  |
| *Lithobius nigripalpis* | 8.48 | 7.72 | 4.47 | 4.37 | 3.23 | 28.27 |  | 7.27 | 6.35 |
| *Lithobius pamukkalensis* |  |  | 1.00 |  |  |  |  |  |  |
| *Pachymerium ferrugineum* |  |  |  | 3.59 |  |  |  |  |  |
| *Schendyla nemorensis* |  |  |  |  |  |  |  |  | 0.40 |
| *Scolopendra cretica* | 4.51 | 18.72 | 13.94 | 19.37 | 44.52 | 4.56 | 1.67 | 0.89 | 0.93 |
| *Scutigera coleoptrata* | 12.31 | 1.63 | 8.27 | 12.95 |  | 3.26 | 9.54 | 2.65 | 7.45 |
| Total | 31.58 | 33.95 | 33.69 | 43.93 | 53.30 | 66.35 | 12.14 | 25.69 | 25.66 |
